# Lactylation in cardiovascular disease: mechanisms and therapeutic potential

**DOI:** 10.1038/s41419-026-08977-7

**Published:** 2026-06-26

**Authors:** Chen-Xi Feng, Shao-Cong Wang, Xin-Zhe Chen, Ying-Hui Li, Chun Zhao, Mei-Hua Zhang, Kun Wang, Lu-Yu Zhou

**Affiliations:** 1 Department of Pharmacy, College of Biology, Hunan University, Changsha, Hunan China; 2 Key Laboratory of Maternal and Fetal Medicine of National Health Commission of China, Institute of Chronic Diseases, Shandong Provincial Maternal and Child Health Care Hospital Affiliated to Qingdao University, Jinan, Shandong China

**Keywords:** Valvular disease, Molecular biology

## Abstract

Lactate, traditionally regarded as a metabolic byproduct, has recently been recognized as a critical signaling molecule and an epigenetic regulator through the post-translational modification known as lactylation. Lactylation links cellular metabolic states to gene transcription by modifying lysine residues on histone and non-histone proteins, thereby influencing inflammation, immune responses, fibrosis, angiogenesis and energy metabolism. Emerging evidence demonstrates that lactylation plays pivotal roles in cardiovascular diseases (CVDs), including atherosclerosis, ischemic heart disease, heart failure and cardiomyopathies. Lactylation orchestrates macrophage polarization, endothelial dysfunction, vascular smooth muscle cell phenotypic switching, cardiomyocyte metabolic remodeling and immune cell activation, ultimately shaping disease initiation and progression. Moreover, lactylation exhibits context-dependent dual effects: promoting reparative processes such as inflammation resolution and tissue healing, while also driving pathological remodeling, fibrosis, mitochondrial dysfunction and maladaptive immune activation. Advances in analytical techniques including LC-MS/MS, lactylation-specific antibodies, and metabolic flux analysis have enabled comprehensive profiling of lactylation dynamics in cardiovascular tissues. Given its central role in metabolic–epigenetic crosstalk, targeting lactylation and its regulatory enzymes represents a promising therapeutic strategy for modulating cardiac injury, remodeling and repair. This review summarizes the molecular basis, cellular functions, pathological implications and therapeutic potential and limited of lactylation in CVDs, providing new insights into its translational value for cardiovascular medicine.

## Introduction

Lactate, once regarded merely as a byproduct of anaerobic glycolysis, has emerged as a critical regulator in cellular metabolism and signaling. Traditionally viewed as a waste product accumulating during low oxygen conditions, lactate is now recognized as a key intermediary in metabolic communication between cells, tissues, and organs [1]. The lactate shuttle hypothesis illustrates its dual role as both an energy substrate and a signaling molecule, facilitating the transport of metabolic intermediates across various cellular environments. Moreover, lactate participates in gene regulation through post-translational modifications, particularly lactylation—the modification of histones and non-histone proteins—which influences gene transcription and cellular responses [2]. This discovery links metabolic activity to epigenetic regulation, enhancing our understanding of lactate’s role in health and disease.

Lactylation, specifically the modification of lysine residues on histones, has been implicated in regulating cellular processes such as inflammation, immune responses, and tissue repair. These processes are crucial in the context of diseases like cancer, cardiovascular diseases, and metabolic disorders. Furthermore, the potential to manipulate lactate metabolism and lactylation presents novel therapeutic strategies, especially for conditions where metabolic dysregulation contributes to disease progression, such as myocardial infarction and heart failure [3]. The role of lactate in gene expression modulation underscores its significance in both normal physiology and disease pathophysiology, offering new avenues for therapeutic intervention.

The primary objective of this article is to provide a comprehensive review of the molecular mechanisms, biological functions, and therapeutic potential of CVDs. Cardiovascular diseases, including coronary artery disease, myocardial infarction, and heart failure, are often associated with metabolic dysfunctions and inflammatory responses. Lactate, through its role in glycolysis and lactylation, is implicated in various pathological processes, such as inflammation, fibrosis, and angiogenesis. This article will first delve into the process of lactylation, exploring how lactate modifies proteins and influences cellular pathways in the heart and vasculature. It will then discuss the regulatory effects of lactylation on gene expression and its involvement in the progression of CVDs, including how it modulates metabolic pathways and immune responses. Finally, the article will examine the potential of targeting lactylation as a therapeutic strategy to manage cardiovascular diseases. By synthesizing current research, the article aims to highlight lactylation as a promising avenue for future therapeutic interventions and provide insights into the role of metabolic reprogramming in the context of CVDs.

## Molecular basis of lactylation

**Fig. 1:**
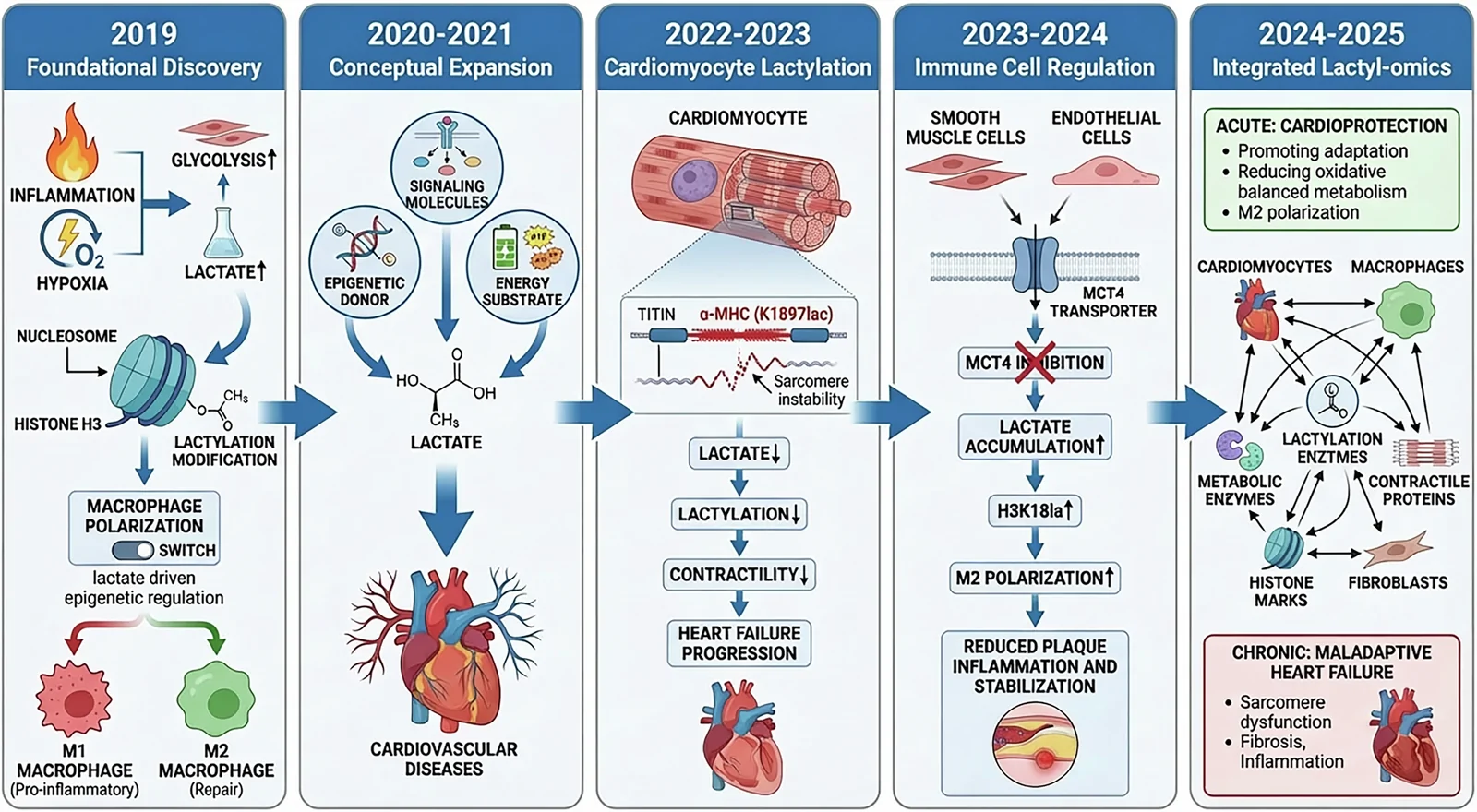
Overview of the temporal and conceptual development of lactylation research. This figure summarizes the progression of lactylation research from its initial discovery to its current integration into cardiovascular, metabolic, and immune regulatory networks.

Lactylation is a novel post-translational modification (PTM) that plays a critical role in connecting cellular metabolism with gene expression regulation [4]. It is primarily regulated by lactate, a product of glycolysis, and involves the covalent attachment of a lactyl group to the lysine residues on histone and non-histone proteins. This modification significantly influences various cellular processes, including inflammation, metabolic reprogramming, immune responses, and gene transcription [1]. The discovery of lactylation as an epigenetic modification has provided new insights into how metabolic changes can influence cellular behavior and contribute to the development and progression of diseases such as cancer, cardiovascular disorders, and metabolic syndromes. Lactylation serves as a bridge between cellular metabolism and epigenetic regulation, providing a mechanistic link between a cell’s metabolic state and its transcriptional activity. Figure 1 summarizes the temporal development of lactylation research, from its initial identification to its integration into cardiovascular and immune regulatory networks.

Lactate, a key metabolite in cellular energy production, is primarily generated through the process of glycolysis, where glucose is broken down into pyruvate. Under aerobic conditions, pyruvate enters the mitochondria for oxidative phosphorylation, producing ATP. However, under hypoxic or anaerobic conditions, or when cells experience increased energy demands, pyruvate is converted to lactate instead. This conversion is catalyzed by lactate dehydrogenase (LDH) and serves as a mechanism to regenerate NAD+ from NADH, allowing glycolysis to continue and provide energy in the form of ATP. Lactate production is especially prominent in rapidly proliferating cells, such as those found in tumors, immune cells, and tissues undergoing inflammation or injury [5]. Lactate was once thought to be a metabolic waste product, but recent research has revealed that it is a versatile molecule that functions as both an energy substrate and a signaling molecule [6]. Lactate can be transported between cells and tissues via monocarboxylate transporters (MCTs), allowing it to serve as a metabolic intermediate that coordinates cellular activities across different organs. Disruptions in lactate transport and metabolism are implicated in various diseases, highlighting the importance of maintaining lactate homeostasis for cellular function and tissue health [7].

**Fig. 2:**
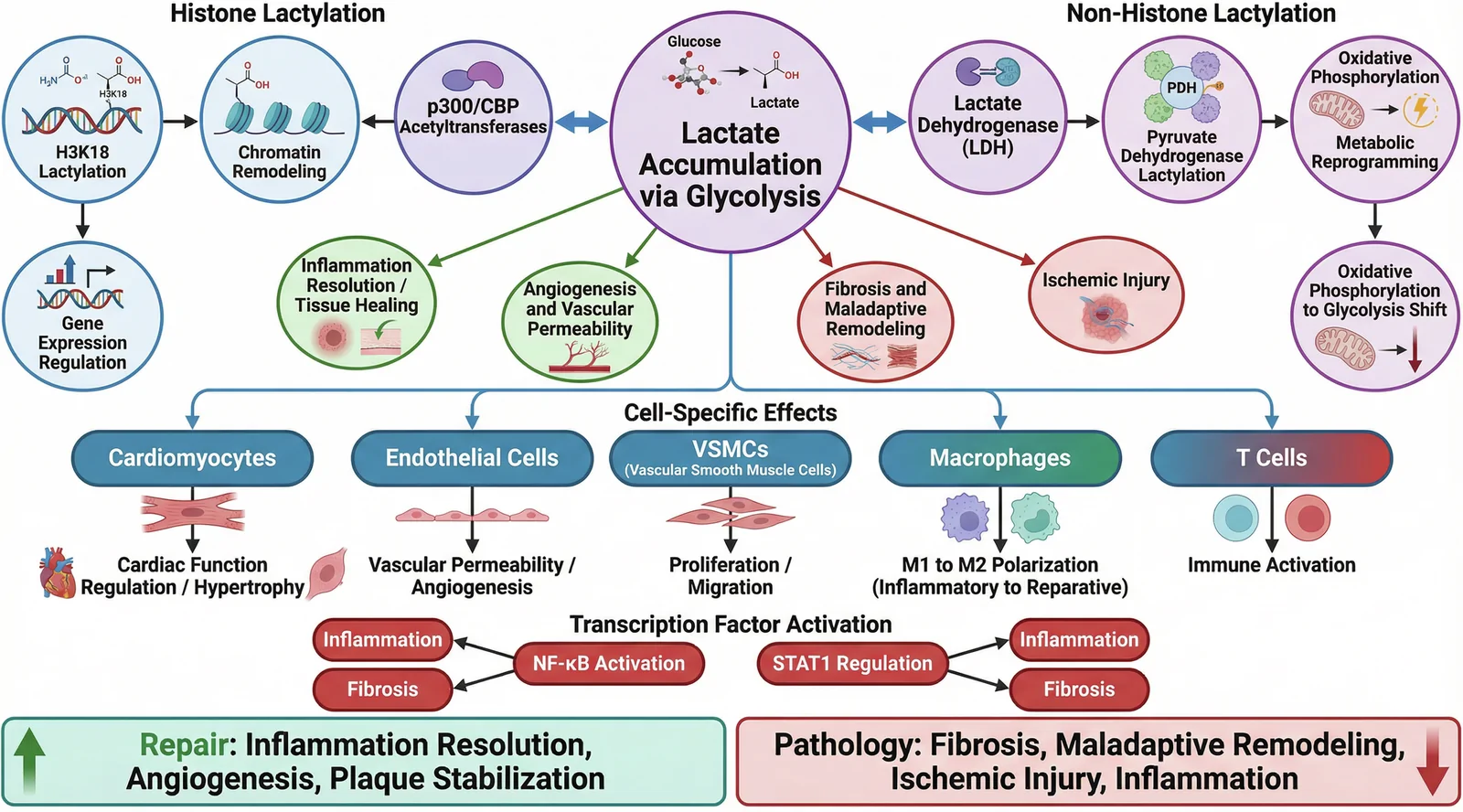
Major pathways and biological consequences of histone and non-histone lactylation. This figure illustrates how glycolysis-derived lactate contributes to histone and non-histone lactylation and how these processes influence gene expression, inflammation, angiogenesis, fibrosis, metabolic reprogramming, and tissue repair.

Histone lactylation, the addition of a lactyl group to lysine residues on histones, directly impacts the chromatin structure and gene expression. This modification has been shown to alter the interaction between histones and DNA, making the chromatin more accessible to the transcriptional machinery, thus promoting the expression of specific genes [8]. The mechanism of histone lactylation involves the enzymatic transfer of a lactyl group from lactyl-CoA, which is produced from lactate, to lysine residues on histone proteins. This reaction is catalyzed by acetyltransferases such as p300 and CREB-binding protein (CBP). These enzymes act as “writers” of histone lactylation, and their activity is influenced by the cellular metabolic state [9]. Histone lactylation has been observed in response to various stress conditions, including hypoxia, metabolic reprogramming, and infection, where increased lactate levels trigger the modification. The lactylation of histone residues, such as histone H3K18 lactylation (H3K18la), is associated with the activation of genes involved in inflammation, immune responses, and tissue repair [10]. This dynamic modification enables cells to adapt to changing metabolic environments by regulating the transcription of key genes involved in cellular survival and stress response [11]. As summarized in Fig. 2, glycolysis-derived lactate contributes to both histone and non-histone lactylation, thereby influencing inflammation, angiogenesis, fibrosis, metabolic reprogramming, and tissue repair.

In addition to enzymatic lactylation, non-enzymatic mechanisms also contribute to lactylation, particularly through the interaction between lactate and glutathione. This non-enzymatic pathway leads to the formation of lactoylglutathione (LGSH), which can react spontaneously with lysine residues on proteins, including histones, resulting in lactylation. This form of lactylation provides a rapid and reversible means of regulating protein function in response to metabolic changes [12]. Unlike the enzymatic pathway, which is tightly regulated by specific enzymes, non-enzymatic lactylation can occur more freely in response to lactate accumulation and metabolic shifts. The presence of LGSH in the cell allows lactate to modify proteins without the need for specific enzyme activity, further highlighting lactate’s role as a signaling molecule capable of affecting gene expression and cellular function across different contexts [1].

Lactylation is not limited to histones but also extends to non-histone proteins involved in critical cellular processes. Non-histone lactylation influences proteins involved in metabolism, DNA repair, and cell signaling. For instance, lactylation of pyruvate dehydrogenase (PDH), a key enzyme in the transition from glycolysis to oxidative phosphorylation, can regulate the cell’s metabolic phenotype by promoting glycolysis over oxidative metabolism. This modification allows cells to adapt to varying energy demands under different conditions, such as during hypoxia or inflammation [13]. Additionally, lactylation of DNA repair proteins like NBS1 and MRE11 has been shown to stabilize repair complexes and enhance the DNA damage response. By modulating the activity of these proteins, lactylation helps maintain genomic stability and facilitates cellular adaptation to environmental stressors, such as DNA damage and oxidative stress [11]. This broader view is also reflected in Fig. 2, which highlights that lactylation should be considered not only as a chromatin-associated modification but also as a regulator of metabolism-dependent cellular phenotypes.

In cardiovascular diseases, lactate and lactylation play significant roles in disease pathogenesis, particularly in the context of myocardial infarction (MI), heart failure (HF), and atherosclerosis. In these diseases, metabolic reprogramming shifts cellular metabolism towards increased glycolysis, leading to lactate accumulation and the activation of lactylation pathways [14]. In the heart, the accumulation of lactate during ischemia or heart failure results in the lactylation of key proteins involved in cardiac remodeling, inflammation, and fibrosis. For example, the lactylation of histone proteins like H3K18la and H4K12la regulates genes that contribute to fibrotic scarring and inflammatory responses, exacerbating myocardial injury (Table1). In addition to histone modifications, lactylation also influences non-histone proteins involved in cellular signaling and immune responses, contributing to the pathological processes associated with heart disease [3].

**Table 1:** Functional consequences and regulatory mechanisms of lysine lactylation in human diseases.

Modification type	Target site	Target protein type	Effect	Associated disease/pathology	Regulatory mechanism
Lactylation	H3K18la	Histone	Promotes transcription, increases expression of inflammatory genes (e.g., IL-6, CCL2)	Myocardial aging, inflammation	Activates NF-κB, promotes senescence-associated secretory phenotype (SASP)
Lactylation	H4K12la	Histone	Promotes expression of glycolysis-related genes	Myocardial dysfunction, heart failure	Co-activation by p300
Lactylation	H3K4la	Histone	Promotes gene transcription related to cell cycle control	Cancer progression	Regulated by p300
Lactylation	PKM2	Non-histone	Decreases enzyme activity, promotes lactate accumulation	Cancer (e.g., bladder cancer, lung cancer)	Affects glycolytic pathway, feedback regulation of lactylation
Lactylation	P53	Non-histone	Increases expression of genes involved in cell cycle and senescence	Myocardial aging, fibrosis	Regulates transcription, promotes senescence
Lactylation	VEGF	Non-histone	Promotes angiogenesis	Cardiovascular diseases, tumors	Regulates angiogenesis via lactylation
Lactylation	MCT1	Non-histone	Promotes lactate uptake and transport	Metabolic diseases	Facilitates lactate transport between glycolytic and oxidative cells

Lactylation is particularly important in regulating immune responses. In immune cells such as macrophages, lactylation drives the expression of genes that modulate inflammation and immune activation. During inflammation or infection, lactate accumulation in immune cells promotes the expression of pro-inflammatory cytokines and immune-related genes through lactylation [15]. This modification helps coordinate the immune response by regulating the polarization of macrophages from a pro-inflammatory M1 phenotype to a reparative M2 phenotype [16]. Lactylation thus acts as a crucial regulator of immune homeostasis, helping to balance inflammation and tissue repair. In diseases like atherosclerosis and MI, lactylation promotes the resolution of inflammation by activating anti-inflammatory and pro-angiogenic genes, which are essential for tissue repair and healing after injury [7, 17].

In conclusion, lactylation is a dynamic and reversible post-translational modification that links cellular metabolism with gene expression regulation. It is directly influenced by lactate levels, which are modulated by metabolic states and cellular energy demands [18]. Lactylation affects histones and non-histone proteins, regulating gene expression, immune responses, metabolic processes, and cell signaling. This makes lactylation a key player in various diseases, including cardiovascular disorders, cancer, and metabolic diseases [19]. Understanding the molecular mechanisms of lactylation provides valuable insights into how metabolic changes can affect cellular function and contribute to disease pathogenesis. Furthermore, lactylation presents an exciting target for therapeutic intervention, offering new opportunities for modulating metabolic and epigenetic pathways in disease treatment [20].

## Lactylation in cardiovascular cell biology

**Fig. 3:**
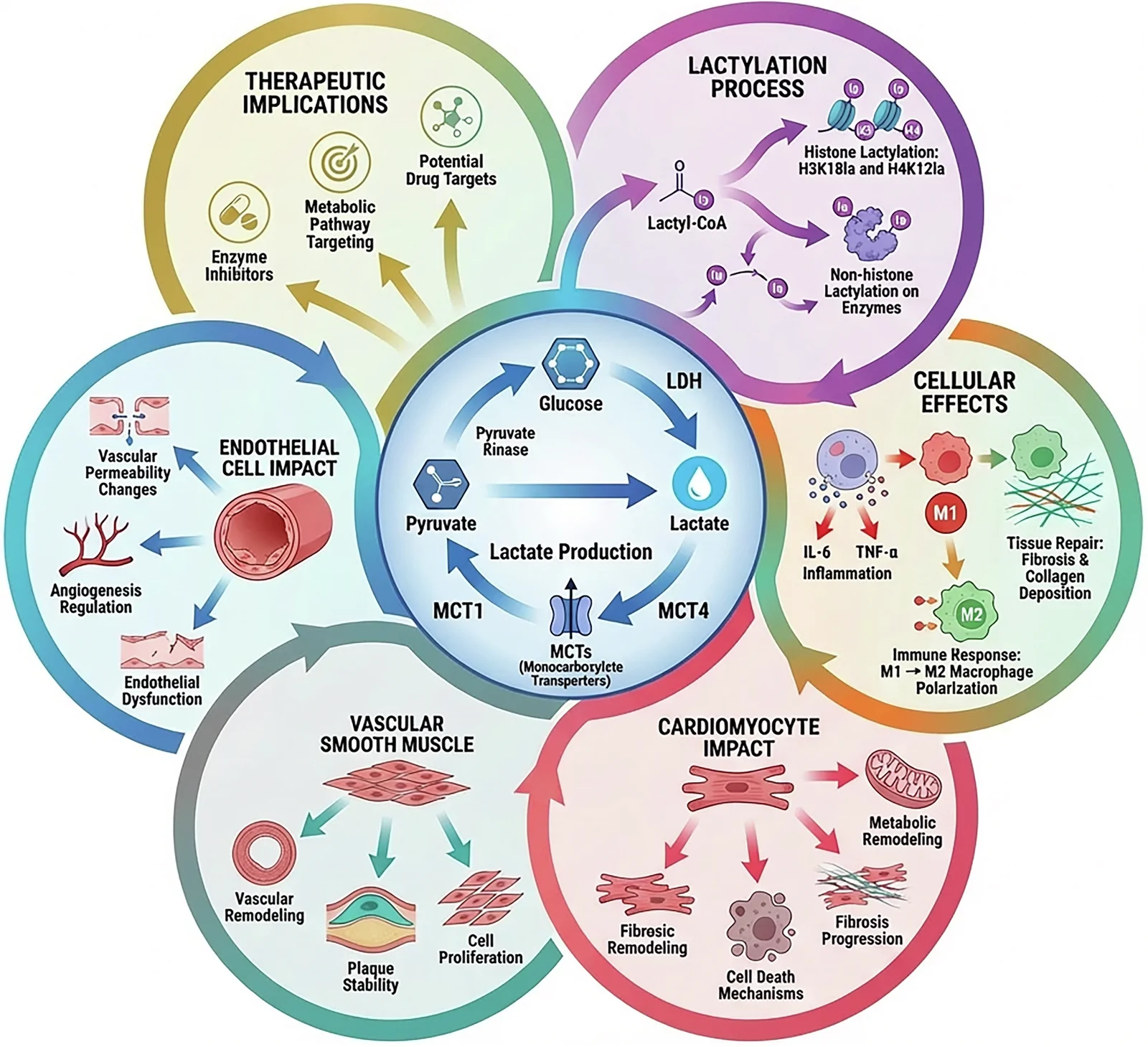
Cell-specific cardiovascular effects and therapeutic relevance of lactylation. This figure summarizes the impact of lactylation on cardiomyocytes, endothelial cells, vascular smooth muscle cells, and immune cells, and highlights their relevance to remodeling, inflammation, fibrosis, and potential therapeutic targeting.

Lactylation links metabolic processes with cellular functions, allowing cells to adapt to changes in their metabolic environment [21]. In the context of cardiovascular biology, lactylation plays a critical role in regulating the function of key cell types such as cardiomyocytes, endothelial cells, vascular smooth muscle cells, macrophages, and T cells [22]. By influencing cellular metabolism, gene expression, and protein function, lactylation contributes to cardiovascular health and disease, particularly in response to metabolic shifts, inflammation, and ischemia [23]. As lactylation modifies proteins involved in energy production, signaling pathways, and cellular interactions, it serves as a crucial mechanism for adapting to stress, injury, and disease, influencing both normal and pathological cardiovascular processes [24]. Figure 3 summarizes the major cardiovascular cell types affected by lactylation and highlights how these effects converge on angiogenesis, inflammation, fibrosis, remodeling, and therapeutic targeting.

### Cardiomyocytes

In cardiomyocytes, lactylation significantly impacts energy metabolism and contractility [25]. The heart is a highly metabolic organ that constantly requires energy to maintain its function [26]. Under conditions of increased demand or stress, such as exercise, ischemia, or heart failure, cardiomyocytes shift their metabolism toward glycolysis, resulting in increased lactate production and lactylation of proteins involved in this metabolic pathway [27]. Lactylation regulates enzymes such as pyruvate kinase and lactate dehydrogenase, which are key players in glycolysis, helping maintain ATP production under low oxygen conditions. Furthermore, lactylation plays a role in cardiac hypertrophy by modifying transcription factors and histones that drive the expression of genes involved in cell enlargement and contractility [28]. In heart failure, altered lactylation can impair calcium handling proteins like SERCA2a and RyR2, leading to reduced contractility and contributing to the progression of heart failure [29].

### Endothelial cells

Endothelial cells, which line the blood vessels, are essential for maintaining vascular integrity and regulating processes such as blood flow, vascular permeability, and immune cell adhesion [30]. Lactylation plays a significant role in endothelial cell function, particularly during inflammatory conditions. Under stress or injury, endothelial cells shift their metabolism toward glycolysis, leading to lactate accumulation and subsequent lactylation of key regulatory proteins [31]. This modification affects the structure and function of endothelial cell junctions, which control vascular permeability. Lactylation of proteins involved in tight junction formation, such as ZO-1 and occludin, can increase vascular permeability, contributing to edema and tissue leakage in diseases like atherosclerosis and heart failure [32]. Additionally, lactylation regulates angiogenesis by modifying proteins such as VEGF and HIF-1α, which are critical for endothelial cell migration, proliferation, and tube formation [33]. This modification is essential for tissue repair and ischemic responses but may also contribute to pathological angiogenesis in conditions like diabetic retinopathy and cancer. Lactylation also modulates the endothelial inflammatory response, influencing the expression of adhesion molecules like ICAM-1 and VCAM-1, which facilitate the recruitment of immune cells to sites of vascular injury or plaque formation, further promoting chronic inflammation and plaque instability [34].

### Vascular smooth muscle cells (VSMCs)

Vascular smooth muscle cells are crucial for regulating blood vessel tone and structural integrity. Lactylation regulates VSMCs function, particularly during vascular remodeling in response to injury or disease [35]. Under pathological conditions such as hypertension or atherosclerosis, VSMCs transition from a contractile phenotype to a synthetic one, characterized by increased proliferation and migration [36]. Lactylation modulates key transcription factors such as SRF (serum response factor) and myocardin, which are involved in VSMCs differentiation and function. By modifying these factors, lactylation enhances VSMCs proliferation and migration in response to growth factors like PDGF and TGF-β [37]. Additionally, lactylation influences the contractile properties of VSMCs by modifying proteins involved in calcium signaling, such as myosin and actin, which are essential for smooth muscle contraction. In pathological vascular remodeling, altered lactylation can contribute to the formation of fibrous caps in atherosclerotic plaques, increasing plaque instability and the risk of plaque rupture [38]. By modulating VSMCs behavior, lactylation plays a key role in vascular remodeling and the development of cardiovascular diseases such as atherosclerosis, restenosis, and hypertension.

### Macrophages

Macrophages are central to the immune response in cardiovascular diseases, where they contribute to inflammation, tissue repair, and remodeling. Lactylation influences macrophage activation and polarization, which are key processes in the development and progression of cardiovascular diseases such as atherosclerosis [39]. Under inflammatory conditions, macrophages can polarize into either a pro-inflammatory (M1) or anti-inflammatory (M2) phenotype. Lactylation regulates this polarization by modifying transcription factors such as NF-κB and STAT1, which control the expression of cytokines and other inflammatory mediators [40]. Inflammatory macrophages, characterized by an M1 phenotype, produce pro-inflammatory cytokines like TNF-α and IL-1β, which drive the chronic inflammation seen in atherosclerosis. Lactylation also affects macrophage phagocytosis and the resolution of inflammation. Impaired lactylation can lead to dysfunction in the clearance of apoptotic cells, promoting plaque instability and contributing to plaque rupture [23, 41]. Additionally, lactylation regulates the secretion of matrix metalloproteinases (MMPs), which degrade the extracellular matrix and destabilize atherosclerotic plaques, increasing the risk of thrombosis and cardiovascular events [28].

### T Cells

T cells play a crucial role in adaptive immunity and are involved in the pathogenesis of various cardiovascular diseases, particularly autoimmune disorders like vasculitis and cardiac allograft rejection [42, 43]. Lactylation regulates T cell activation, differentiation, and metabolic reprogramming, influencing both immune responses and tissue damage in the cardiovascular system. Upon activation, T cells shift their metabolism from oxidative phosphorylation to glycolysis, increasing lactate production and promoting lactylation of proteins involved in T cell signaling [44]. Lactylation modifies transcription factors such as NFAT (nuclear factor of activated T cells) and AP-1, which are critical for T cell activation, differentiation, and cytokine production. In cardiovascular autoimmune diseases, lactylation affects the differentiation of T cells into inflammatory subsets, such as Th1 and Th17 cells, which contribute to tissue damage [33, 45]. Furthermore, lactylation influences the formation and function of memory T cells, which are involved in the persistence of chronic inflammation in cardiovascular tissues. By modulating T cell responses, lactylation plays a critical role in immune-mediated cardiovascular diseases and represents a potential target for therapeutic intervention [38]. Collectively, these cell-specific responses provide the mechanistic basis for the disease-associated roles of lactylation discussed in the following sections and are schematically integrated in Fig. 3.

## Lactylation in cardiovascular diseases

### Atherosclerosis and the role of lactylation

Atherosclerosis (AS) is a chronic inflammatory disease characterized by the accumulation of lipids, inflammatory cells, and extracellular matrix in the arterial walls, leading to the formation of plaques that can narrow or obstruct blood vessels [46]. Over time, these plaques may become unstable, rupture, and cause life-threatening cardiovascular events, such as heart attacks and strokes. Macrophages play a central role in the development and progression of atherosclerosis, as they are involved in both the initiation of inflammation and the resolution of plaque damage [47, 48]. Macrophages within atherosclerotic lesions exhibit plasticity, with the ability to polarize into distinct phenotypes depending on the local microenvironment. The pro-inflammatory M1 macrophages exacerbate plaque instability, while the M2 macrophages, often referred to as reparative macrophages, contribute to tissue repair and plaque stabilization [49, 50].

Recent research has highlighted the role of metabolic reprogramming and epigenetic modifications in regulating macrophage function in atherosclerosis. In inflammatory environments within atherosclerotic plaques, macrophages undergo metabolic reprogramming characterized by enhanced aerobic glycolysis (the “Warburg-like effect”), which results in increased intracellular lactate production through lactate dehydrogenase-mediated conversion of pyruvate to lactate. Accumulated lactate can serve not only as a metabolic by-product but also as a substrate for the generation of lactyl-CoA, which enables lysine lactylation of histone proteins via acyl-transferase enzymes such as p300. One of the key post-translational modifications that has emerged in this context is lactylation. This modification is particularly relevant in atherosclerosis, where macrophages undergo metabolic shifts toward increased glycolysis, leading to elevated lactate production within the plaque microenvironment [51, 52].

For example, studies have shown that in atherosclerosis models, the accumulation of lactate due to metabolic reprogramming in macrophages enhances histone lactylation and promotes a shift toward the M2 phenotype [53]. Mechanistically, lactate-derived histone lactylation occurs on specific lysine residues of histone H3 (such as H3K18la), which relaxes chromatin structure and facilitates transcriptional activation of genes associated with reparative macrophage functions. In ApoE-deficient mice, which are commonly used to model atherosclerosis, exercise-induced lactate accumulation has been found to increase the lactylation of histones in macrophages, leading to enhanced expression of reparative genes like Arg1, characteristic of the M2 macrophage phenotype. This epigenetic activation enhances transcription factor accessibility at the Arg1 promoter and promotes anti-inflammatory gene programs that suppress NF-κB-driven inflammatory signaling. This transition is essential for reducing inflammation and stabilizing plaques [54].

Additionally, a study on the methyl-CpG-binding protein 2 (MeCP2) in endothelial cells within atherosclerotic plaques revealed that increased lactate levels could enhance the lactylation of MeCP2. MeCP2 is a chromatin-associated transcriptional regulator that typically binds methylated CpG islands and modulates gene transcription by recruiting co-repressor complexes. Lactylation of MeCP2 alters its chromatin-binding affinity and transcriptional regulatory activity. This lactylation suppressed the expression of pro-inflammatory genes, such as IL-6, and promoted the expression of reparative factors, thereby contributing to plaque stabilization and reducing the risk of rupture [49].

The accumulation of lactate in atherosclerotic plaques acts not only as an energy source but also as an important signaling molecule that regulates macrophage behavior through histone lactylation. Through this mechanism, lactylation serves as a metabolic–epigenetic bridge linking glycolytic metabolism to transcriptional reprogramming in macrophages. Lactylation can influence the expression of genes involved in macrophage polarization, promoting the transition from the pro-inflammatory M1 phenotype to the anti-inflammatory M2 phenotype [51, 55]. This process is associated with increased expression of genes involved in tissue remodeling, collagen synthesis, and efferocytosis, which collectively enhance plaque stability. M2 macrophages facilitate tissue repair, remove apoptotic cells, and promote collagen deposition in the fibrous cap [56].

Exercise-induced lactate has also been shown to enhance lactylation in macrophages and endothelial cells within atherosclerotic plaques. Regular exercise increases circulating lactate levels, which can be transported into vascular tissues through MCTs [57]. Once internalized, lactate can directly participate in histone lactylation, thereby activating anti-inflammatory gene expression programs in vascular immune cells. Exercise has been demonstrated to attenuate atherosclerosis progression by improving plaque stability, reducing inflammation, and increasing the collagen content in the fibrous cap, which helps prevent plaque rupture and reduces the risk of cardiovascular events.

In conclusion, lactylation is a crucial mechanism linking metabolic reprogramming to macrophage polarization in atherosclerosis. By directly modifying histone proteins and transcriptional regulators, lactylation reshapes gene expression programs that favor reparative macrophage phenotypes and suppress inflammatory signaling. By promoting the transition toward a reparative phenotype, lactylation contributes to plaque stabilization and the resolution of inflammation, making it a promising therapeutic target for atherosclerosis [58]. Understanding the molecular mechanisms of lactylation-mediated macrophage reprogramming may open new avenues for the development of therapies aimed at modulating macrophage epigenetic states to halt or even reverse disease progression.

### Ischemic heart disease

Ischemic heart disease (IHD), including conditions like MI and HF, arises due to impaired blood flow to the heart, often from atherosclerotic plaque buildup in the coronary arteries [29]. The blockage or rupture of these plaques leads to acute ischemia, followed by reperfusion injury (IRI) when blood flow is restored, causing further myocardial damage [59, 60].

Macrophages play a pivotal role in both the initiation of inflammation and the resolution of tissue damage in the heart. They contribute to inflammatory responses following ischemic injury but also participate in repair mechanisms, such as tissue regeneration and fibrosis [59, 61]. Following myocardial infarction, circulating monocytes are recruited to the infarcted myocardium through chemokine signaling pathways such as the CCL2-CCR2 axis. These monocytes differentiate into macrophages that dynamically transition between inflammatory and reparative phenotypes during different phases of cardiac remodeling. The polarization of macrophages into distinct phenotypes, particularly the pro-inflammatory M1 phenotype and the reparative M2 phenotype, is therefore crucial in determining the outcome of IHD. The balance between these phenotypes influences the extent of myocardial injury and repair [62].

One of the primary roles of lactylation in IHD is in regulating macrophage polarization. Recent studies suggest that lactate accumulation during ischemic injury functions not only as a metabolic by-product but also as an epigenetic signaling molecule that regulates gene transcription through protein lactylation. In a recent study by Xu et al., metabolic reprogramming in macrophages during ischemic stress led to increased glycolytic flux and intracellular lactate accumulation, which subsequently enhanced histone lysine lactylation [63, 64]. Mechanistically, lactate-derived lactyl groups are transferred to lysine residues of histones by acetyltransferases such as p300, generating histone lactylation marks (e.g., H3K18la) that promote chromatin relaxation and transcriptional activation of specific genes. This lactylation promotes the shift of macrophages toward the reparative M2 phenotype, which is essential for tissue repair and the resolution of inflammation. For instance, in ApoE−/− mice, a model of atherosclerosis and ischemia, exercise-induced lactate accumulation increased histone lactylation in macrophages. These genes encode proteins that suppress inflammatory cytokine production, promote extracellular matrix remodeling, and facilitate tissue repair.

In addition to regulating macrophage polarization, lactylation also modulates the activity and stability of transcription factors involved in cardiac remodeling. In a 2025 study by Zhang et al., lactate accumulation in ischemic heart tissue was shown to induce lactylation of Snail1, a transcription factor that regulates endothelial-to-mesenchymal transition (EndoMT) and fibrosis. Lactylation of Snail1 occurs on lysine residues that influence its ubiquitination and proteasomal degradation. By preventing ubiquitin-mediated degradation, lactylation enhances Snail1 protein stability and transcriptional activity. As a result, Snail1 activates downstream profibrotic genes, including those involved in extracellular matrix deposition and fibroblast activation, thereby promoting fibrosis within the infarcted myocardium. EndoMT driven by stabilized Snail1 converts endothelial cells into mesenchymal-like cells that contribute to myofibroblast accumulation and pathological cardiac remodeling. However, the same study demonstrated that inhibition of Snail1 or blockade of lactate transporters could attenuate these effects, highlighting the therapeutic potential of targeting lactylation pathways to reduce fibrosis and prevent maladaptive remodeling following MI [31].

In addition to macrophages, lactylation also affects the behavior of cardiomyocytes and endothelial cells in ischemic heart disease. During myocardial ischemia-reperfusion, metabolic reprogramming toward glycolysis leads to rapid lactate accumulation within the ischemic microenvironment. This elevated lactate can enter immune cells and endothelial cells via MCTs, thereby promoting intracellular lactylation events. In studies of myocardial ischemia-reperfusion injury, lactate-induced lactylation of key proteins involved in mitochondrial function and calcium homeostasis has been implicated in exacerbating myocardial injury [65]. For example, Zhang et al. demonstrated that lactate accumulation in neutrophils induced histone H3K18 lactylation at the promoter region of mitochondrial calcium uptake protein MICU3, thereby enhancing its transcription. Elevated MICU3 interacts with voltage-dependent anion channel 1 (VDAC1) to promote the formation of mitochondria-associated membranes (MAMs), which facilitate excessive mitochondrial Ca²⁺ uptake. This calcium overload disrupts mitochondrial membrane potential, increases ROS production, and triggers mitochondrial dysfunction. Furthermore, lactylation also stabilized MICU3 protein through interactions with AASR1, amplifying its effects on mitochondrial calcium influx. These events ultimately promoted the formation of neutrophil extracellular traps (NETs), which aggravated endothelial cell injury and microvascular dysfunction in myocardial ischemia-reperfusion injury.

Given the dual role of lactylation in both promoting myocardial repair and exacerbating inflammation or fibrosis, it represents a promising therapeutic target in ischemic heart disease [66]. Therapeutic modulation of lactylation may therefore require context-dependent strategies aimed at enhancing beneficial reparative signaling while suppressing pathological remodeling pathways. For instance, exercise has been shown to induce moderate lactate accumulation that promotes histone lactylation in macrophages and endothelial cells. This controlled lactylation response may activate anti-inflammatory and pro-angiogenic gene programs that stabilize atherosclerotic plaques and improve endothelial function. In preclinical models, exercise-induced lactylation improved plaque stability and reduced the risk of rupture by shifting macrophage polarization toward the M2 phenotype and preserving endothelial integrity [67].

Furthermore, strategies aimed at inhibiting pathological lactylation events, such as those driving fibrosis or excessive inflammatory activation, may help prevent maladaptive cardiac remodeling in IHD. Potential therapeutic approaches include inhibition of lactate transporters (such as MCT1/4), modulation of lactylation “writer” enzymes such as p300, or enhancement of delactylation processes mediated by histone deacetylases. By selectively targeting these regulatory mechanisms, it may be possible to attenuate the detrimental effects of lactylation while preserving its beneficial roles in tissue repair and immune regulation [68].

### Heart failure

HF is a complex clinical syndrome characterized by the heart’s inability to pump sufficient blood to meet the body’s metabolic demands. This condition leads to impaired tissue perfusion, resulting in multi-organ dysfunction [69]. HF is often associated with a range of underlying pathologies, including myocardial injury, neurohormonal dysregulation, and metabolic disturbances. Despite significant advances in its treatment, HF remains a major global health issue, with high mortality rates and limited therapeutic options [70, 71]. Recent research has begun to focus on the metabolic and epigenetic factors that contribute to the pathogenesis of HF, with lactylation emerging as a key PTM involved in the disease [72].

In HF, the metabolic shift from oxidative phosphorylation to anaerobic glycolysis increases lactate production, leading to elevated levels of lactate in the heart. Recent studies indicate that excess intracellular lactate can serve as a substrate for lysine lactylation, a post-translational modification in which lactyl groups derived from lactyl-CoA are covalently attached to lysine residues of histone and non-histone proteins. This process is dynamically regulated by metabolic enzymes acting as “writers” and “erasers”, including acyltransferases that add lactyl groups and deacylases such as histone deacetylases (HDACs) and sirtuins that remove them, thereby linking cellular metabolic status to epigenetic and signaling regulation. This metabolic reprogramming is accompanied by lactylation of proteins, which modulates cellular functions, including inflammation, metabolic regulation, and fibrosis [73, 74].

One of the key effects of lactylation in HF is its role in exacerbating the inflammatory response. For example, lactylation of BRD4, a bromodomain protein, has been shown to enhance immune cell activation and infiltration into the heart. Mechanistically, BRD4 functions as an epigenetic reader that recognizes acylated lysine residues on histones and recruits transcriptional machinery to promoters of inflammatory genes. Lactylation of BRD4 enhances its chromatin-binding affinity and promotes the transcription of pro-inflammatory mediators through activation of NF-κB-dependent signaling pathways. This results in increased expression of cytokines such as IL-6, TNF-α, and MCP-1, which drive immune cell recruitment and sustain chronic inflammation in the failing myocardium. This promotes the release of pro-inflammatory cytokines, which contribute to the ongoing inflammation and fibrosis observed in HF [21]. In addition, lactylation of proteins involved in the regulation of cardiac fibrosis, such as TGF-β1, further exacerbates myocardial remodeling. TGF-β1 is a central regulator of fibrotic signaling that activates the canonical SMAD2/3 pathway. Lactylation of TGF-β1-associated regulatory proteins enhances downstream SMAD phosphorylation and transcriptional activation of extracellular matrix genes, including COL1A1 and fibronectin, thereby promoting fibroblast activation and extracellular matrix accumulation. The modification of these proteins by lactate enhances their activity, driving the pathological changes that impair cardiac function [75].

Furthermore, lactylation impacts cardiac metabolism by modifying enzymes involved in fatty acid oxidation and glycolysis. In the failing heart, this modification shifts the metabolic profile of cardiomyocytes, favoring glycolysis over fatty acid oxidation. This metabolic shift results in an energy crisis within the heart, which contributes to the dysfunction of cardiomyocytes and exacerbates the symptoms of HF [76]. Mechanistically, lysine lactylation can alter enzyme conformation and catalytic efficiency, thereby modulating substrate affinity and metabolic flux. Specifically, lactylation alters the function of enzymes such as CPT1 and FASN, inhibiting fatty acid metabolism and promoting glycolysis, which further accelerates the metabolic dysfunction in HF [77]. For instance, modification of CPT1 may impair mitochondrial fatty-acid transport, while lactylation-induced activation of lipogenic enzymes such as FASN can promote lipid metabolic imbalance, ultimately reducing ATP production and worsening myocardial energetic insufficiency.

Lactylation also plays a role in promoting cardiac fibrosis, a critical factor in the progression of HF. The accumulation of lactate in the myocardium triggers the activation of fibroblasts and their differentiation into myofibroblasts, leading to the excessive deposition of extracellular matrix components and the subsequent stiffening of the heart tissue [78]. This process is partly mediated through lactylation-dependent transcriptional activation of profibrotic genes and enhanced responsiveness of fibroblasts to TGF-β signaling, which accelerates collagen synthesis and myofibroblast differentiation. This process is mediated by lactylation of key proteins involved in the fibrotic response, such as TGF-β1, which upregulates collagen production and accelerates myocardial fibrosis.

In addition to its effects on inflammation and fibrosis, lactylation is involved in the regulation of apoptosis in cardiomyocytes. Studies have shown that lactylation can modify proteins involved in apoptosis pathways, contributing to cell death and exacerbating the damage to the heart muscle [79]. For example, lactylation may influence the balance between pro-apoptotic and anti-apoptotic factors by modulating signaling pathways such as the Bcl-2/Bax axis and caspase activation cascades, thereby promoting mitochondrial dysfunction and cardiomyocyte apoptosis. This process is particularly important in the context of acute myocardial infarction, where lactate accumulation induces lactylation of apoptotic regulators, promoting cardiomyocyte loss and tissue damage [80].

Recent studies have also focused on targeting the enzymes involved in lactylation as potential therapeutic strategies for HF. Inhibitors of HDACs and sirtuins, which regulate the removal of lactyl groups, have been shown to reduce lactylation and alleviate the severity of HF in animal models [72]. For example, inhibiting the activity of HDACs has been demonstrated to decrease lactylation levels in the heart, reduce fibrosis, and improve cardiac function. Additionally, the identification of lactylation-related genes (LRGs) through bioinformatics approaches has provided new biomarkers for diagnosing HF and assessing its progression. The upregulation of genes such as BRD4 in response to lactylation has been linked to immune cell infiltration and inflammation, offering a potential target for therapeutic intervention [21].

### Cardiac hypertrophy and cardiomyopathy

Hypertrophic cardiomyopathy (HCM) and ischemic cardiomyopathy (ICM) are two major forms of heart disease, both associated with significant myocardial remodeling, dysfunction, and impaired heart function. HCM is the most common inherited cardiac disease, characterized by the thickening of the myocardium, particularly the left ventricle, leading to obstructive and non-obstructive forms [81, 82]. In contrast, ICM is primarily caused by coronary artery disease, resulting in ischemia, myocardial infarction, and ischemia-reperfusion injury, all of which contribute to heart failure and other serious cardiovascular complications [83]. Both conditions involve complex metabolic reprogramming, with significant alterations in energy metabolism, protein function, and gene expression, leading to progressive cardiac dysfunction. Recent research has highlighted the role of lactylation in modulating these pathological processes in both HCM and ICM [84].

In HCM, the heart muscle undergoes hypertrophic remodeling due to increased mechanical stress, often triggered by genetic mutations or prolonged pressure overload from hypertension. This hypertrophic response is initially adaptive, helping the heart maintain adequate cardiac output [85, 86]. However, sustained pressure overload results in maladaptive remodeling, contributing to heart failure, arrhythmias, and impaired relaxation of the heart muscle. While genetic factors and altered signaling pathways are well known to drive HCM, emerging evidence suggests that metabolic reprogramming, particularly lactylation, plays a critical role in the progression of hypertrophy [87].

In HCM, lactate accumulation—resulting from increased glycolysis and impaired mitochondrial oxidative phosphorylation—promotes histone lactylation, particularly at the H3K18 site [85]. H3K18 lactylation (H3K18la) promotes chromatin relaxation at promoter regions of hypertrophy-associated genes and enhances the binding of transcription factors such as MEF2 and NFAT, thereby facilitating transcriptional activation of fetal cardiac gene programs. These include β-myosin heavy chain (β-MHC), atrial natriuretic peptide (ANP), and brain natriuretic peptide (BNP), which are classical markers of pathological hypertrophic remodeling. For example, a study using a Myh7 V878A gene mutation in mice—a model for HCM—demonstrated that lactate accumulation increased the expression of H3K18la, promoting hypertrophic gene expression and fibrosis markers like collagen type I [88].

In addition to histones, non-histone proteins involved in energy metabolism also undergo lactylation. Proteins like Acads and Acaa2, which are involved in fatty acid oxidation, were found to be lactylated in HCM models, indicating a shift towards glycolysis and away from oxidative metabolism [86]. These enzymes play key roles in mitochondrial β-oxidation and the tricarboxylic acid (TCA) cycle. Lactylation of these metabolic enzymes may alter their catalytic activity or structural stability, thereby reducing fatty-acid-derived ATP production and reinforcing a metabolic switch toward glycolysis. This metabolic remodeling is characteristic of hypertrophic hearts, where ATP generation becomes less efficient but more rapid, supporting sustained cardiomyocyte growth and biosynthetic processes. Consequently, lactylation-mediated inhibition of fatty acid oxidation contributes to energy imbalance and exacerbates maladaptive myocardial hypertrophy [81].

ICM, resulting from ischemic injury, involves metabolic derangements due to a lack of oxygen and nutrients in the myocardium. During myocardial infarction or ischemia-reperfusion injury, glycolysis becomes the primary energy pathway, leading to the accumulation of lactate in the heart muscle [89, 90].

Lactylation has been shown to exacerbate myocardial damage in ICM [91]. Under hypoxic conditions, stabilization of hypoxia-inducible factor-1α (HIF-1α) enhances glycolytic gene expression, further increasing lactate production. The elevated intracellular lactate pool serves as a substrate for p300-mediated histone lactylation, thereby linking hypoxic metabolic stress with epigenetic gene regulation in cardiomyocytes. For example, one study demonstrated that lactate-induced lactylation of H3K18la in ischemic cardiomyocytes activated genes involved in cell survival and repair, such as VEGF and IL-10, but this was not sufficient to fully counteract the damage caused by the ischemic event [67, 92].

Furthermore, lactylation in ICM has been linked to changes in macrophage function. Lactate accumulation during ischemia can influence immune cell polarization, particularly shifting macrophages towards a pro-inflammatory phenotype. This shift exacerbates the inflammatory response and impedes the myocardial repair process, contributing to further tissue damage. Studies have shown that lactylation affects proteins involved in immune responses, such as NLRP3 inflammasome components, which play a pivotal role in the inflammatory cascade following ischemic injury [83].

In addition to its effects on immune cells, lactate-induced lactylation also disrupts mitochondrial function in cardiomyocytes. This is particularly relevant in ischemic injury, where mitochondrial dysfunction is a hallmark of cell death. Emerging evidence suggests that lactylation of mitochondrial proteins may interfere with oxidative phosphorylation by altering the activity of key enzymes within the electron transport chain and the TCA cycle. These modifications can promote excessive reactive oxygen species (ROS) generation, impair ATP synthesis, and trigger mitochondrial permeability transition pore opening, ultimately leading to cardiomyocyte apoptosis or necrosis during reperfusion injury [84].

The role of lactylation in both HCM and ICM underscores its complex involvement in regulating myocardial metabolism, gene expression, and cellular signaling. On the one hand, lactylation in HCM drives maladaptive hypertrophic remodeling by promoting glycolytic metabolism and activating hypertrophic gene expression [93, 94]. On the other hand, in ICM, lactate-induced lactylation exacerbates ischemic injury by modulating gene expression related to inflammation, fibrosis, and cell death. While lactylation may initially promote adaptive repair mechanisms in response to ischemic injury, persistent lactylation and its effects on metabolic and inflammatory pathways ultimately contribute to the progression of heart disease [95].

## Detection and analytical techniques

**Fig. 4:**
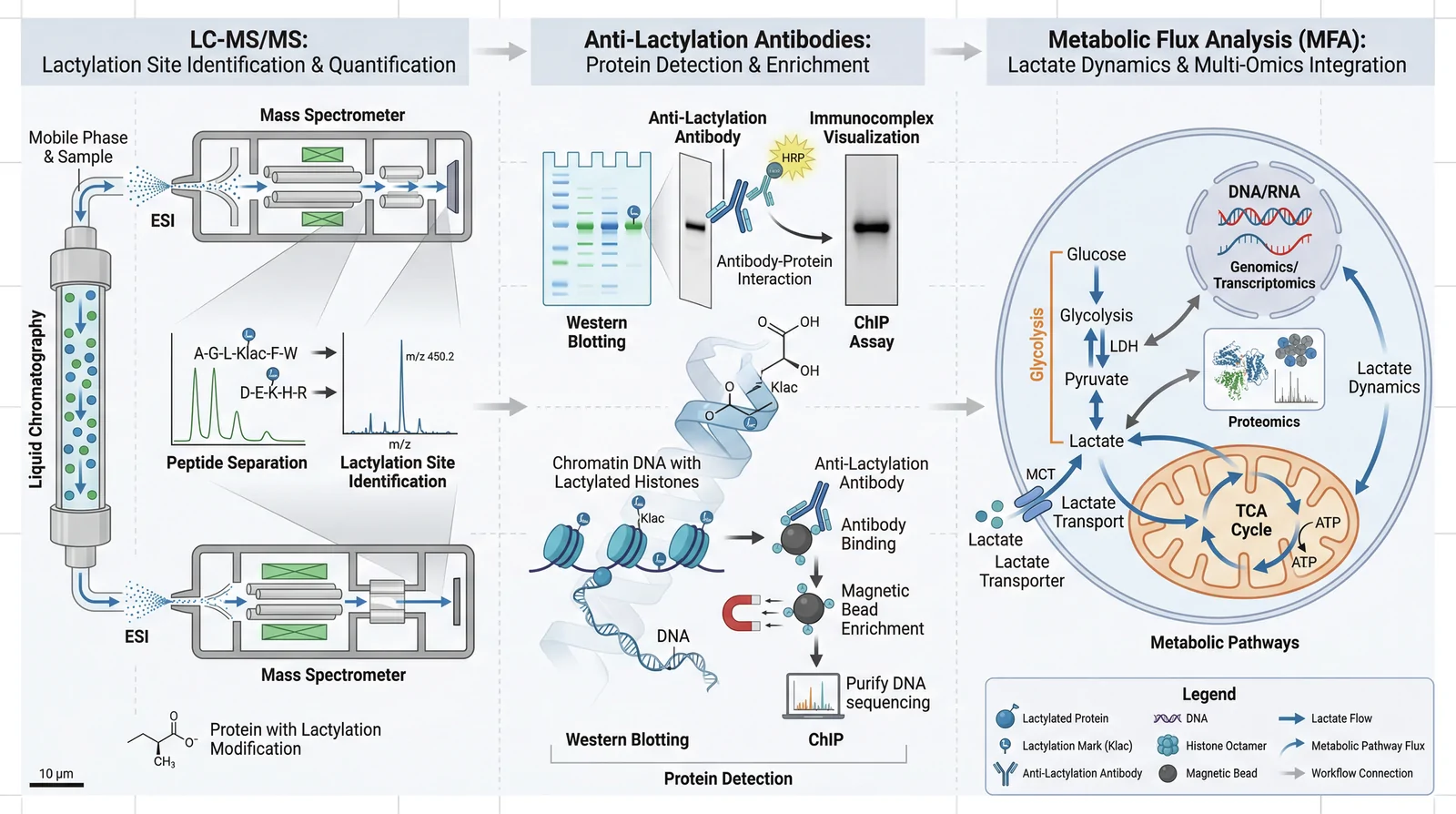
Representative analytical approaches for lactylation research. This figure summarizes three major methodological categories used to investigate lactylation: LC-MS/MS for site identification and quantification, antibody-based assays for detection and enrichment, and metabolic flux analysis for integrating lactate dynamics with multi-omics data.

The detection and analysis of lactylation have become crucial for understanding its role in cellular function and disease progression. Recent advancements in analytical techniques, including Liquid chromatography-tandem mass spectrometry /mass spectrometry(LC-MS/MS), the development of anti-lactylation antibodies, and metabolic flux analysis (MFA), have significantly improved our ability to study lactylation in complex biological systems [96]. These methodologies, often combined with integrative omics approaches, provide a comprehensive framework for exploring the dynamic roles of lactylation in cellular metabolism and its potential as a therapeutic target. Figure 4 summarizes the three major analytical strategies discussed in this section, namely LC-MS/MS for site identification, antibody-based assays for validation and enrichment, and metabolic flux analysis for linking lactylation to lactate dynamics and pathway activity.

### Liquid chromatography-tandem mass spectrometry/mass spectrometry

LC-MS/MS has become one of the key techniques for analyzing lactylation in proteins. By coupling high-resolution mass spectrometers with liquid chromatography, LC-MS/MS enables the accurate identification and quantification of lactylation modifications in complex biological samples. The advantage of mass spectrometry lies in its high sensitivity, which allows for the detection of lactylation sites and enables the quantification of lactylated peptides at low abundance. Through selective enrichment of lactylated peptides, LC-MS/MS significantly improves the detection rate of lactylation modifications in proteins [97].

In particular, LC-MS/MS, when combined with gel electrophoresis separation and enzymatic digestion, allows for the systematic identification of lactylation sites within complex protein samples. For example, in cardiac cells, researchers have used LC-MS/MS to analyze lactylation sites on key proteins involved in energy metabolism and mitochondrial function. These analyses have not only revealed the role of lactate in cellular metabolism but also demonstrated that lactylation is closely linked to various pathological conditions, including cardiac hypertrophy and neurodegenerative diseases [98].

Additionally, LC-MS/MS can be integrated with isotope labeling techniques to track the dynamic changes of lactate and its flow through metabolic pathways. In Fig. 4, this workflow is represented as the primary approach for identifying lactylation sites and quantifying lactylated peptides in complex samples.This combination provides further insights into the biological functions and mechanisms of lactate in different biological processes.

### Development and application of anti-lactylation antibodies

Anti-lactylation antibodies play a critical role in the detection of lactylation. These antibodies are widely used in techniques such as Western blotting (WB) and chromatin immunoprecipitation (ChIP) to confirm the presence and extent of lactylation modifications in proteins. By using lactylation-specific antibodies, researchers can effectively identify and quantify lactylation, particularly in histones and non-histone proteins [54]. Notably, histone lactylation on residues like H3K18 and H4K12 has been a major focus of research. Anti-lactylation antibodies allow for the specific identification of these modifications, helping to elucidate their impact on chromatin structure, gene expression, and cellular functions [99, 100].

In ChIP assays, lactylation antibodies are employed to enrich chromatin fragments that carry lactylation modifications, thus revealing the influence of lactylation on specific gene regulatory regions. Studies have shown that lactylation can modulate the binding of transcription factors, thereby regulating the expression of genes associated with metabolism, immune responses, and cell proliferation [101]. In cardiovascular diseases and atherosclerosis, WB and ChIP techniques have been extensively applied to uncover how lactylation regulates cellular functions in various pathological contexts. Notably,VSMCs senescence, lactylation has been shown to activate the senescence-associated secretory phenotype (SASP), promoting pathological changes [38, 102]. As illustrated in Fig. 4, antibody-based approaches complement MS-based detection by enabling protein-level validation, enrichment of lactylated targets, and locus-specific analysis in chromatin-associated studies.

### Metabolic flux analysis and integrative omics research

Metabolic flux analysis (MFA) is a powerful tool for studying the movement of metabolites within cellular networks. In lactylation research, MFA can help trace the metabolic sources of lactate and its dynamic changes within cells. For example, in disease models such as cardiac hypertrophy and cancer, MFA has been used to track the generation, transport, and utilization of lactate, revealing how lactate influences cellular energy status and function through metabolic reprogramming.

When combined with other omics technologies, MFA provides a more comprehensive metabolic map. By integrating metabolic flux data with genomics, transcriptomics, and proteomics, researchers can gain deeper insights into how lactylation modifications regulate cellular behavior through metabolic pathways. For instance, lactate accumulation is closely associated with mitochondrial dysfunction, and lactylation, as an important epigenetic modification, regulates the expression of genes and proteins related to mitochondrial function [103, 104]. Figure 4 further emphasizes that MFA is particularly useful for integrating lactate transport, glycolytic activity, and multi-omics datasets, thereby connecting metabolic flux with lactylation-dependent cellular responses.

As the role of lactylation in cellular metabolism and disease regulation becomes increasingly recognized, advancements in LC-MS/MS, the development of anti-lactylation antibodies, and the use of metabolic flux analysis and integrative omics have provided invaluable tools for studying lactylation. These techniques have enabled a deeper understanding of how lactylation influences gene expression, cellular metabolism, and function across a range of diseases. As research into lactylation continues to expand, these technologies will likely play an even more pivotal role in the diagnosis and treatment of diseases such as cardiovascular disorders, neurodegenerative diseases, and cancer. The future of lactylation research holds promise for the identification of novel therapeutic strategies targeting lactylation pathways to mitigate disease progression.

## Treatment and limitations

Lactylation plays a dual role in cardiovascular diseases, exhibiting both protective and maladaptive effects. On one hand, lactylation contributes to tissue repair and recovery, particularly in acute cardiovascular events such as myocardial infarction. It promotes M2 macrophage polarization and activates repair-associated genes, such as Lrg1 and IL-10, which mitigate inflammation and enhance angiogenesis, thereby facilitating tissue repair and cardiac remodeling post-infarction [105, 106]. This protective effect underscores the adaptive role of lactylation in maintaining cardiac homeostasis and promoting recovery under stress conditions. Additionally, lactate accumulation in the heart, often observed in hypoxic or high-energy-demand states, can act as a critical energy source, regulated by lactylation, to support cellular function under adverse conditions [107, 108].

However, the role of lactylation in chronic cardiovascular diseases presents a more complex picture. In heart failure and atherosclerosis, lactate accumulation, while indicative of metabolic reprogramming, may lead to detrimental outcomes. Excessive lactate accumulation in heart failure is associated with impaired myocardial contractility, and lactylation may hinder the activation of repair genes, exacerbating the disease process [88]. Similarly, in atherosclerosis, lactylation has been shown to promote smooth muscle cell proliferation and calcification, driving maladaptive vascular remodeling [107]. These findings highlight that lactylation’s effects are context-dependent, with protective roles under certain conditions, but potentially harmful effects in chronic disease states.

Additionally, lactylation and acetylation, both occurring at lysine residues, represent key post-translational modifications with distinct effects on cellular functions, particularly in cardiovascular diseases. These two modifications often compete for the same lysine sites, leading to a complex regulatory balance that influences gene expression and cellular responses [109, 110]. Lactylation, typically associated with metabolic reprogramming and increased glycolytic flux, promotes a pro-inflammatory environment by enhancing the expression of genes involved in inflammation and endothelial dysfunction. This modification has been implicated in the progression of atherosclerosis and other cardiovascular conditions. In contrast, acetylation is generally linked to anti-inflammatory responses and gene activation that support cellular repair and homeostasis. Acetylation of histone residues such as H3K9, for example, helps suppress inflammation and promotes endothelial cell function, thereby protecting against vascular damage. The competition between these modifications—lactylation promoting inflammation and acetylation counteracting it—plays a critical role in determining the progression or resolution of cardiovascular pathologies, with a proper balance being essential for maintaining vascular health and preventing disease progression [48].

Despite the promising therapeutic potential of targeting lactylation, significant challenges remain. The absence of specific lactylation inhibitors and the complexity of lactylation regulation hinder the development of targeted therapies. Lactylation is regulated by a variety of “writer” enzymes (such as p300 and KAT8) and “eraser” enzymes (like HDAC1-3 and SIRT1), whose activities are influenced by specific cell types and pathological conditions. This makes the development of precise lactylation-targeted therapies particularly challenging, as it requires understanding the dynamic regulation of lactylation across different tissues and disease stages [7, 107]. Moreover, lactylation is not confined to histones; it also occurs on non-histone proteins, adding another layer of complexity to its functional implications in cellular signaling and metabolism.

Future research should focus on developing selective lactylation modulators that can precisely control lactylation in specific tissues and at appropriate time points. Techniques such as CRISPR/dCas9 for spatiotemporal regulation of lactylation could provide novel therapeutic strategies. Moreover, a deeper understanding of the dynamic regulatory networks of lactylation across various disease contexts is essential for translating lactylation-based interventions into clinical practice. In summary, while lactylation presents a promising avenue for cardiovascular disease therapy, its clinical application requires overcoming significant challenges related to its complex regulation and context-dependent effects.

## Conclusion

In conclusion, lactylation represents a critical post-translational modification that bridges the gap between cellular metabolism and gene expression regulation. This modification, driven by lactate accumulation, has profound implications for the pathogenesis of cardiovascular diseases, including myocardial infarction, heart failure, and atherosclerosis. Lactylation not only influences gene expression through histone modifications but also modulates the function of non-histone proteins involved in key cellular processes such as inflammation, fibrosis, and tissue repair. These modifications enable cells to adapt to shifting metabolic environments, influencing cellular responses to stress, injury, and disease.

In cardiovascular diseases, lactylation plays a dual role—both promoting repair processes and exacerbating pathological conditions such as fibrosis and inflammation. It governs immune cell polarization, metabolic reprogramming, and the remodeling of cardiac and vascular tissues. Notably, lactylation in macrophages contributes to the resolution of inflammation and stabilization of atherosclerotic plaques, while in ischemic heart disease, it exacerbates myocardial injury and impairs recovery through its effects on mitochondrial function and inflammatory responses.

Given the significant role of lactylation in cardiovascular pathology, targeting this modification holds potential as a therapeutic strategy. Interventions aimed at modulating lactate metabolism or lactylation pathways could offer novel approaches to prevent disease progression, enhance tissue repair, and improve clinical outcomes in patients suffering from heart diseases. Further research into the molecular mechanisms underlying lactylation, combined with advances in detection techniques such as mass spectrometry and metabolic flux analysis, will be essential for understanding its full impact and developing targeted therapies to harness its potential in clinical settings.

In summary, lactylation is emerging as a crucial regulator of cardiovascular disease processes, and its therapeutic modulation presents a promising avenue for improving cardiovascular health and addressing unmet clinical needs in heart disease management.

## Data Availability

All data are included in this article.
